# Effect of age-friendly community degree on the cognitive function of older adults

**DOI:** 10.3389/fpsyt.2026.1927936

**Published:** 2026-09-28

**Authors:** Laiyou Li, Ning Sun, Shuangqin Chen, Shuang Yang, Hongyu Li, Qianru Li, Kaiying Zhong, Junxin Yan

**Affiliations:** 1 Ningbo College of Health Sciences, Ningbo, China; 2 College of Landscape Architecture, Northeast Forestry University, Harbin, China

**Keywords:** age-friendly community, cognitive impairment, community, influencing factors, older people

## Abstract

**Introduction:**

The relationship between individual environmental indicators and the physical and mental health of older adults has been widely studied. However, the holistic impact of age-friendly communities on the wellbeing of older adults has not beenclearly elucidated. This study aimed to explore the correlation between the degree of community friendliness and cognitive function of older adults and provide scientific evidence for healthy aging.

**Methods:**

This cross-sectional study enrolled 450 residents aged ≥60 years with cognitive impairment in Ningbo, Zhejiang Province, from June to September 2024. Participants provided personal demographic data, and their community’s age-friendly status and cognitive function were assessed using the Age-Friendly Community (AFC) Evaluation Scale and the Alzheimer’s Disease 8 Questionnaire. A multivariate logistic regression analysis was performed to examine the relationship between demographic characteristics and cognitive function of the older adults.

**Results:**

Among the five dimensions of the AFC evaluation scale, community participation scored the highest at 3.56 ±' 1.21 points. Multivariate linear regression analysis revealed that personal demographic factors (age, sex, education level, economic status, housing conditions, alcohol consumption, and chronic diseases) significantly infiuenced cognitive function (all P < 0.05). Furthermore, residential environment, transportation, and community environment were identified as significant predictors of cognitive impairment (all P < 0.05).

**Discussion:**

Low scores in residential environment, transportation, community environment, and social inclusion in AFC are independent risk factors for cognitive dysfunction. Measures to enhance community age-friendly environments could help improve the cognitive function of older adults.

## Introduction

Age-friendly communities have become a research hotspot under the global aging context. To promote healthy aging, the World Health Organization proposed the concept of age-friendly communities in 2006, which gradually attracted attention from the academic community. In 2020, the National Health Commission and Office of the National Working Committee on Aging issued the “Notice on Carrying Out the Creation of Model National Age-Friendly Communities,” providing a policy basis and fundamental guidance for systematic research on age-friendly communities (1). With population aging, dementia imposes a heavy economic burden and social development pressure in China and worldwide (2). Dementia is characterized by cognitive dysfunction, and early control of risk factors and rational utilization of protective factors can prevent or delay the deterioration of cognitive dysfunction (3). Although the factors influencing cognitive dysfunction have been widely researched, most studies have focused on individual characteristics such as general features, genetic background, and behavioral lifestyle, with less attention on the impact of community environment.

Frailty represents a multidimensional aging-related vulnerability state in older adults. As frailty progresses, older adults face elevated risks of mobility impairment and depressive symptoms. These adverse physical and psychological outcomes, in turn, exacerbate the deterioration of cognitive function (4–6). In China, although many older adults sustain moderate overall health status (5, 7), aging-related health deterioration may trigger a series of adverse consequences, including impaired self-care capacity, reduced social participation, and decreased engagement in recreational activities (8). Such cumulative health losses impose heavy care burdens on individual families and the whole society (9). Therefore, safeguarding the physical and mental health of older adults serves as a core strategy to tackle population aging challenges and advance the construction of a healthy aging society.

The World Health Organization “Global Report on Ageing and Health” (2015) emphasized that environmental factors and individual capabilities jointly promote functional performance for healthy aging (10, 11). The environment encompasses all the external factors that constitute an individual’s life, including natural, social, and supportive components (8). The development of age-friendly communities has gained momentum (12–14). Age-friendly communities focus on the needs and interests of older adults by providing community settings and services tailored to their requirements. These communities encourage older adults to pursue their interests, build connections, and engage actively, thus enhancing their well-being and self-confidence (15). Exploring the relationship between the level of age-friendly communities and the health status of older adults can provide policy guidance and practical foundation for healthy aging. Previous studies have revealed a close correlation between the community environment and older adult health (16), offering a theoretical basis for investigating the relationship between the levels of age-friendly communities and health outcomes.

As defined by the World Health Organization, age-friendly communities refer to supportive residential environments that integrate multiple dimensions including residential facilities, public transportation, ecological landscape, and social inclusion, aiming to accommodate the physical, psychological, and social needs of older adults. The built environment of age-friendly communities may be related to the cognitive function of older adults (17, 18). For example, the use of public transportation can enable older adults to lead intellectually, socially, and physically active lives, which can consequently promote their cognitive function (19). Previous domestic studies have confirmed the positive effects of community environmental factors on cognitive abilities among urban older adults (20, 21). Yanmin and Chunyu (22) further indicated that outdoor environmental design in age-friendly communities facilitates older adults’ social interaction, which serves as a critical protective factor for maintaining cognitive function. Nevertheless, existing relevant studies mostly adopt single or scattered environmental indicators for measurement, lacking comprehensive evaluation based on the systematic dimensional framework of age-friendly communities. Given the ambiguous and fragmented measurement of age-friendly community attributes in current evidence, holistic research regarding the comprehensive impact of multi-dimensional age-friendly community environments on older adults’ cognitive wellbeing remains insufficient, which necessitates further systematic empirical exploration.

This study aimed to investigate the correlation between age-friendly communities and cognitive impairment in older adults, seeking to provide a scientific basis for promoting healthy aging.

## Materials and methods

### Study design

This study employed a correlational cross-sectional design, and questionnaires were used for data collection.

### Setting and sample

Convenience sampling was used to recruit older adults from five communities in Ningbo, China, between June and September 2024. A total of 450 older adults completed the questionnaires with the help of their home caregivers. The inclusion criteria were as follows: 1) caregiving experience for at least 6 months; 2) age >60 years; 3) diagnosis of dementia using the 8-item ascertained dementia (AD8) scale, and 4) conscious and able to voluntarily participate in the study. The exclusion criteria were as follows: 1) severe cognitive impairment; 2) inability to comprehend the questionnaire content; and 3) severe heart, liver, lung, or brain dysfunction.

### Sample size calculation

The study focuses on the correlation between age-friendly communities and cognitive impairment. Multiple linear regression analysis was used to analyze the data. Twenty variables could be entered into the model. Given that the required sample size should be at least 10–15 times the number of variables in the model, a sample of 300 participants was required (23). Based on the 20% missing rate was used for calculation. The total sample required more than 360 participants.

### Data collection

Two investigators underwent similar training for data collection. Data were collected through face-to-face interviews after the participants engaged in scheduled meetings arranged by community managers. The investigators explained the research objectives and methods to the participants and obtained informed consent. Consenting participants received an envelope containing questionnaires. The participants completed the questionnaires immediately upon receipt and placed them in an envelope for collection by the investigators. Each completed questionnaire was assigned a code to ensure anonymity.

### Instruments

Three structured questionnaires were used for data collection. The general health survey for community-dwelling older adults encompassed demographic characteristics (age, sex, education level, marital status, housing conditions, financial status, and community name), health indicators (body mass index, chronic disease count, sleep quality, and self-assessed health status), and behavioral factors (smoking, alcohol consumption, physical activity, and social engagement).

The Chinese version of the Age-Friendly Community Rating Scale assesses whether communities adequately meet the daily living needs and social engagement requirements of older adults. It also evaluates the age friendliness of communities. Based on the original scale, Jixiang et al. (24)conducted localized revisions to make the content and entries more suitable for the Chinese context. The Chinese version of the Age-Friendly Community Evaluation Scale contains 32 items, divided into residential environment, transportation, community environment, community participation, and social healthcare services. The scale comprises five Likert items: 5, 4, 3, 2 and 1 points for “strongly agree,” “agree,” “neutral,” “disagree,” and “strongly disagree,” respectively. The Cronbach’s alpha for the Chinese version scale was 0.972. A higher total score indicates a more friendly community for older adults.

Cognitive function status was assessed using the Ascertain Dementia 8-item Informant Interview (AD8). Developed by the University of Washington, the original English scale demonstrates strong reliability and validity, with Cronbach’s α = 0.78 and test-retest reliability = 0.96, making it a simple yet sensitive tool for early dementia detection (25). The AD8 scale comprises eight assessment items: (1) difficulty in judgment; (2) reduced interest in hobbies or activities; (3) repetitive behaviors; (4) difficulties in learning to use tools; (5) forgetting correct months and years; (6) challenges in handling complex financial matters; (7) trouble remembering scheduled times; and (8) daily cognitive or memory issues. Each item is scored 1 point for “yes (cognitive change exists)” and 0 point for “no”. The total score ranges from 0 to 8. In the present study, the Cronbach’s α coefficient of the Chinese version of the AD8 was 0.87. Participants were asked whether they had experienced cognitive changes over the past few years. Consistent with widely adopted screening criteria, a score ≥2 was defined as cognitive impairment (25).

### Ethical considerations

This study was approved by Ningbo college of health sciences Institutional Review Board (approval no. NBWY-031). All the participants provided written informed consent before participation. Two researchers were responsible for informing the participants, both in writing and orally, about the study purpose and data collection. The participants were informed that participation in the survey was voluntary and that withdrawal from the study would have no negative consequences.

### Data analysis

The study utilized Microsoft Excel for database construction and SPSS 26.0 for statistical analysis, with significance levels set at *P* < 0.05. Descriptive statistics were employed to analyze demographic characteristics and current situation of older adult-friendly community levels. Quantitative data are presented as mean ± standard deviation, whereas categorical data are displayed as frequency (n) with percentage. A stepwise regression model was constructed to examine the influence of personal general characteristics and community age-friendliness on cognitive dysfunction, with *P* < 0.05 indicating statistically significant differences.

## Results

### Participant demographics

A total of 450 questionnaires were distributed, and 420 valid responses were collected, achieving a response rate of 93.3%. The mean age of the participants was 73.5 years (range: 60–94 years), and 60.7% were women. The demographic data showed that 61.2% were married and 30.5% were living alone. Half of the couples lived together, and 92.9% had at least a junior high school education. Further, 67.1% of households had a monthly income of <5,000 yuan. Chronic diseases affected 74.5% of the participants; 35.8% had one condition, 25.8% had two, and 38.4% had three or more conditions. Notably, 62.6% of the participants abstained from smoking, 57.1% avoided alcohol, 66.2% exercised regularly, and 27.8% reported average sleep quality. Social engagement was strong, with 51.9% meeting friends/relatives weekly and 44.8% regularly participating in community activities. However, 31.4% reported infrequent or no participation (**Table 1**).

**Table 1:** Demographic data of older adults (n = 420).

Demographics	N	Percentage
Age (years)		
60–74	280	66.7
≥75	140	33.3
Sex		
Male	165	39.3
Female	255	60.7
Marital status		
Married	257	61.2
Widowed	128	30.5
Other	35	8.3
Residence status		
Couples living together	210	50.0
Living alone	88	21.0
Live with children	110	26.2
Other	12	2.8
Education level		
High school or below	310	73.8
College or above	80	19.1
Other	30	7.1
Monthly income		
≤5000 yuan	282	67.1
5000–10000 yuan	112	26.7
>10000 yuan	26	6.2
Number of children		
0	18	4.3
1	142	33.8
2	197	46.9
3 or more	63	15.0
Do you have a chronic illness?		
Yes	313	74.5
Deny	107	25.5
Number of people with chronic diseases		
1	112	35.8
2	81	25.8
3	120	38.4
Smoking		
Yes	157	37.4
Deny	263	62.6
Alcohol consumption		
Yes	180	42.9
Deny	240	57.1
Physical exercise		
Non-participation	44	10.5
Occasional	98	23.3
Regular participation	278	66.2
Health self-assessment		
Fine	53	12.6
Preferably	67	16.0
Same as	88	21.0
Range	113	26.9
Bad	99	23.5
Sleep quality		
Fine	48	11.4
Preferably	83	19.8
Same as	117	27.8
Range	104	24.8
Bad	68	16.2
Meet friends and family		
Less than monthly/never	72	17.1
Weekly	130	31.0
At least weekly	218	51.9
Community activity participation		
Less than monthly/never	132	31.4
Weekly	100	23.8
At least weekly	188	44.8

### Current situation of older adult-friendly community levels

The scores for the older adult-friendly community scale ranged from 33 to 160 points (average score: 115.26 ± 35.87). The dimensions and their corresponding scores were as follows: residential quality, 10.37 ± 3.66; transportation, 24.63 ± 8.21; community environment, 21.32 ± 7.11; community participation, 21.65 ± 7.21; and social healthcare services, 35.47 ± 10.09. Among all the dimensions, community participation scored the highest at 3.56 ± 1.21 points (**Table 2**).

**Table 2:** Scores of older adult-friendly community.

Project	Number of entries	Full score range	Score	Evenly distributed entries
Older adult-friendly community level	32	33–160	115.26 ± 35.87	3.54 ± 1.13
Residential environment	3	3–15	10.37 ± 3.66	3.53 ± 1.21
Transportation	7	7–35	24.63 ± 8.21	3.55 ± 1.17
Community environment	6	6–30	21.32 ± 7.11	3.52 ± 1.16
Community participation	6	6–30	21.65 ± 7.21	3.56 ± 1.21
Social healthcare services	10	10–50	35.47 ± 10.79	3.54 ± 1.18

### Logistic regression analysis of the influence of basic data on cognitive dysfunction

Logistic regression analysis showed that age, sex, education, economic status, living situation, alcohol consumption, and chronic diseases were independent factors of cognitive dysfunction (**Tables 3**, **4**).

**Table 3:** Assignment table.

Argument	Variable name	Assignment description
Socio-demographic factors		
Age (years)	_X1_	0 = 60–74 years old; 1 = 75 years old and above
Sex	_X2_	1=Male; 0=Female
Education level	_X3_	High school or below as reference variable; college or above (1,0); other (0,1)
Marital status	_X4_	Married as reference variable; widowed (1,0); other (0,1)
Living status	_X5_	Couple living together as reference variable; living alone (1,0,0); living with children (0,1,0); others (0,0,1)
Number of children	_X6_	Assign actual values
Monthly income	_X7_	1=less than 5,000 yuan; 2 = 5000–10000 yuan; 3=more than 10,000 yuan
Behavioral factors		
Smoking	_X8_	1=Yes; 0=No
Alcohol consumption	_X9_	1=Yes; 0=No
Exercise	_X10_	1=Not participating; 2=Occasionally participating; 3=Regularly participating
Social activity	_X11_	1= Less than monthly/never; 2= More than monthly but less than weekly; 3= At least weekly
Health-related factors		
Chronic diseases	_X12_	1=Yes; 0=No
Sleep quality	_X13_	1=Very good; 2=Good; 3=Average; 4=Poor; 5=Very poor
Health condition	_X14_	1=Very good; 2=Good; 3=Average; 4=Poor; 5=Very poor

**Table 4:** Logistic regression analysis of the impact of basic data on cognitive dysfunction.

Variable	B	SE	Waldχ^2^	*P* value	OR(95% CI)
Age	0.041	0.005	73.771	<0.001	1.045 (1.032–1.051)
Sex (Ref.=Female)					
Male	0.149	0.064	4.893	0.024	1.165 (1.018–1.324)
Education level (Ref.=High school or below)					
College or above	−0.102	0.030	10.254	0.001	0.894 (0.850–0.959)
Other	-0.036	0.033	1.187	0.276	0.965 (0.904–1.030)
Monthly income	−0.141	0.038	11.607	<0.001	0.872 (0.802–0.934)
Living status (Ref.=Couple living together)					
Living alone	0.632	0.212	9.225	0.002	1.911 (1.258–2.920)
Living with children	0.324	0.221	2.148	0.143	1.383 (0.896–2.134)
Other	0.185	0.236	0.615	0.433	1.203 (0.757–1.912)
Alcohol consumption (Ref.=No)					
Yes	0.059	0.025	5.352	0.020	1.059 (1.007–1.121)
Chronic diseases (Ref.=No)					
Yes	0.061	0.031	4.389	0.035	1.061 (1.004–1.127)

### Logistic regression analysis of the effect of community age-friendliness on cognitive dysfunction

Using cognitive dysfunction as the dependent variable, logistic regression analysis was performed with the five dimensions of the Alzheimer’s Functional Classification as independent variables, while adjusting for age, sex, education level, economic status, housing conditions, alcohol consumption, and chronic disease. Low scores in residential environment (odds ratio [OR], 0.923 [95% confidence interval, CI 0.876–0.970], transportation (OR, 0.924 [95%CI 0.876–0.970]), and community environment (OR, 0.945 [95% CI 0.892–0.997]) were independent risk factors for cognitive dysfunction (**Table 5**).

**Table 5:** Logistic regression analysis of the effect of community age-friendliness on cognitive dysfunction.

Variable	B	SE	Waldχ^2^	*P* price	OR (95% CI)
Residential environment	−0.078	0.026	8.856	0.003	0.923(0.876 ~ 0.970)
Transportation	−0.076	0.028	7.518	0.006	0.924(0.872 ~ 0.976)
Community environment	−0.054	0.027	3.969	0.045	0.945(0.892 ~ 0.997)
Community participation	−0.002	0.027	0.004	0.947	0.997(0.935 ~ 1.051)
Social healthcare services	−0.039	0.028	2.041	0.152	0.958(0.917 ~ 1.017)

## Discussion

### Basic health and behavioral characteristics of community-dwelling older adults

This study analyzed the health status and behavioral characteristics of community-dwelling older adults and further explored their associations with individual cognitive function. In the current sample, 75.8% of older adults were diagnosed with chronic diseases, and 62.6% exhibited multimorbidity, which aligns with the prevalent health status of older adult populations in China reported in previous studies (26, 27). Accumulating evidence indicates that multimorbidity can trigger persistent systemic inflammatory responses and progressive physical functional decline, which serve as crucial biological risk factors for cognitive impairment and accelerated cognitive aging in older adults. Additionally, 66.2% of participants in this study maintained regular physical exercise. Consistent with prior empirical findings, sustained physical activity contributes to reduced systemic inflammation, improved cerebral blood perfusion, and enhanced brain neuroplasticity, thereby exerting significant protective effects on cognitive function. Such healthy lifestyle behaviors can effectively buffer the adverse impacts of physiological risk factors on cognition and interact with community environmental conditions to jointly shape the cognitive health of older adults.

### Current status of older adult-friendly community perception

Distinct from existing studies that predominantly adopt objective built-environment indicators (e.g., green space coverage, road accessibility, and public facility density) to evaluate community livability, the present study assessed the level of age-friendly communities based on older adults’ subjective perception and comprehensive experience evaluation. Subjective community evaluation reflects individuals’ psychological adaptation, environmental satisfaction, and perceived livability, which differs substantially from objective community indicators in terms of action mechanisms and health-related effects. Objective community attributes primarily influence physical health through direct environmental exposure, whereas subjective community perception further affects individual psychological stress levels, social participation willingness, and daily behavioral patterns, ultimately generating differentiated impacts on the cognitive health of older adults.

Based on the convenience sample included in this study, the overall perceived score of age-friendly communities was at a moderate level (115.26 ± 35.87), with prominent dimensional developmental imbalance across evaluation domains. The community participation dimension yielded the highest score, indicating that older adults generally perceived sufficient organizational support for various community cultural, educational, and recreational activities. These community activities effectively enrich the spiritual and cultural life of older adults and strengthen their individual social connections and community belonging. Nevertheless, the community environment dimension presented a relatively lower score, suggesting that older adults reported suboptimal subjective experience regarding community ecological environment, residential comfort, and overall environmental livability (28). Importantly, due to the inherent limitations of convenience sampling, the findings of this study only reflect the characteristics of the surveyed population and cannot be generalized to other regions or broader elderly groups. The unbalanced developmental status of multiple community dimensions identified in this study reveals existing deficiencies in age-friendly community construction from the subjective resident perspective and provides targeted optimization directions for future community environmental upgrading.

### Individual influencing factors of cognitive dysfunction in older adults

Consistent with the conclusions of previous epidemiological and empirical studies, this study verified that individual demographic, socioeconomic, and health-related factors are core influencing factors for cognitive function among older adults (29–31). The regression results demonstrated that advanced age, male sex, lower educational attainment, insufficient monthly economic income, living with children, regular alcohol consumption, and chronic disease prevalence are independent risk factors for cognitive dysfunction in community-dwelling older adults. Aging is recognized as the most stable non-modifiable risk factor for cognitive decline, as progressive neuronal loss and brain atrophy inevitably elevate the risk of mild cognitive impairment and Alzheimer’s disease (29). Older adults with lower education levels possess limited cognitive reserve capacity, while those with poor socioeconomic status endure long-term life pressure; these adverse conditions accelerate biological aging processes and exacerbate cognitive deterioration (30, 32). Furthermore, chronic diseases and unhealthy drinking behaviors aggravate chronic inflammation and oxidative stress, causing sustained damage to neuronal structure and function and further impairing cognitive performance (31). These inherent individual risk factors constitute the fundamental basis of cognitive dysfunction in older adults and are difficult to reverse in the short term, which highlights the critical value of modifiable external community environmental intervention for cognitive health protection.

### Association between subjective age-friendly community perception and cognitive function

Focusing on the core relationship between age-friendly community and cognitive function, this study found that older adults’ subjective perceptions of residential, transportation, and community environments independently protect against cognitive impairment, supplementing existing evidence in this field. Most prior studies have validated the cognitive benefits of objective community environmental indicators (33, 34). Further controlling for individual demographic, socioeconomic and health confounding factors, the current study innovatively confirms that subjective age-friendly community evaluation exerts unique protective effects on cognitive function, independent of objective community conditions.

Mechanistically, favorable subjective environmental perception alleviates older adults’ chronic psychological stress and negative emotions, reduces cortisol-related neurotoxicity and inflammatory responses, and slows hippocampal atrophy and cognitive deterioration (35). Additionally, positive environmental experience promotes outdoor mobility and social engagement, further protecting cognitive function through behavioral and social pathways.

Importantly, this study also revealed meaningful negative results. Despite a high score in the community participation dimension, no significant association was observed between community participation and cognitive function. This suggests that mere enrichment of community recreational and cultural activities fails to improve cognitive outcomes, as the potential benefits of social participation are limited by suboptimal hardware conditions, inconvenient transportation and poor environmental experience. Such dimensional imbalance of age-friendly communities restricts the health value of community social resources, which has been insufficiently addressed in previous research. Compared with objective environmental indicators, subjective community perception is more closely tied to individual psychological experience and daily behaviors, providing more targeted cognitive protection. For older adults with unmodifiable individual risk factors, optimizing age-friendly community environments to improve subjective environmental experience is an effective population-level strategy to delay cognitive aging.

### Limitations

First, we used convenience samples and self-reports; thus, common method variance may have biased our results. Therefore, a longitudinal multisource study can be conducted to test the causal effects of these variables. Second, the data were obtained from only one city in China, meaning the results may not be generalizable to other cities with different economic levels and aging structures. Therefore, a larger sampling range covering multiple regions can be used to verify the proposed model. Third, this cross-sectional design cannot clarify the temporal causal sequence between age-friendly community indicators and cognitive function; future long-term longitudinal tracking research is required to reveal dynamic mutual relationships.

## Conclusions

This study found a moderate overall level of age-friendly community construction with obvious dimensional disparities, in which community participation performed better, whereas residential environment, transportation and community ecology remained weak areas. Multiple individual characteristics including age, education, economic status, living arrangement and chronic diseases were associated with older adults’ cognitive function. Importantly, residential environment, transportation infrastructure and community environment dimensions of age-friendly communities were independent influencing factors of cognitive dysfunction. Since many individual risk factors are difficult to modify in the short term, targeted optimization of age-friendly community environments is a promising intervention approach to protect cognitive health. It is suggested to build comprehensive, inclusive and service-oriented age-friendly communities by upgrading housing, transportation and ecological facilities, enriching social activities and constructing an age-inclusive atmosphere, so as to delay cognitive decline and promote healthy aging among older adults.

## Data Availability

The raw data supporting the conclusions of this article will be made available by the authors, without undue reservation.
